# MINIMAL CHANGE DISEASE IN PEOPLE LIVING WITH HIV: A CASE REPORT AND REVIEW OF THE LITERATURE

**DOI:** 10.21010/Ajidv19i1.9

**Published:** 2024-10-25

**Authors:** SARAGIH Restuti Hidayani, NASUTION Syafrizal, PANE Jamaluddin, JAYA Fandy Ong

**Affiliations:** 1 Division of Tropical Medicine and Infectious Diseases, Department of Internal Medicine, Faculty of Medicine, Universitas Sumatera Utara, Medan, Indonesia; 2 Division of Nephrology and Hypertension, Department of Internal Medicine, Faculty of Medicine, Universitas Sumatera Utara, Medan, Indonesia; 3 Department of Pathology, Faculty of Medicine, Universitas Sumatera Utara, Medan, Indonesia; 4 Adam Malik General Hospital, Medan, Indonesia; 5 Faculty of Medicine, Universitas Sumatera Utara, Medan, Indonesia

**Keywords:** *AIDS*, *corticosteroid*, *HIV infection*, *minimal change disease*

## Abstract

**Background:**

Various glomerular diseases are associated with human immunodeficiency virus (HIV) infection. However, the incidence of minimal change nephrotic syndrome has scarcely been reported.

**Materials and Method:**

We describe a patient with a stage 4 HIV infection complaining of swelling in his face, hands, feet, genitals and an enlarged abdomen. Urinalysis revealed +3 proteins with normal urine sediment, and 2.1 g protein was found in the 24-hour analysis. Renal ultrasound showed bilateral glomerulopathies with hyperechoic and cortical thickening, a normal kidney size, and ascites. Kidney biopsy revealed acute tubular injury without HIV-associated nephropathy (HIVAN) features. The patient was treated with salt restriction, diuretics, captopril, methylprednisolone, and combined ART for 2 weeks and showed clinical improvement.

**Results:**

Two weeks after the remission, the patient came to the outpatient department with a history of a 3-day cough with rust-colored sputum, fever, malaise, and shortness of breath. The lung auscultation revealed bilateral rhonchi and the chest x-ray result suggesting pneumonia. The patient was diagnosed with sepsis associated with healthcare-associated pneumonia but was not willing to be hospitalized and passed away at home. This study is limited to single-case nature and the possibility of sampling error.

**Conclusion:**

However, this case encourages further study in the field of HIV-associated renal diseases in providing clear recommendation in the management in special population.

## Introduction

Significant progress in treatment using antiretroviral therapy (ART) has increased the quality of life and life expectancy of people living with HIV (PLHIV). However, the development of chronic disease is inevitable. Kidney disease is one of the comorbidities commonly found in PLHIV (Bookholane *et al.*, 2020). Renal complications in PLHIV include acute kidney injury, chronic kidney injury, HIV-associated nephropathy (HIVAN), immune complex-associated kidney disease, and drug nephrotoxicity (Silva Junior *et al.*, 2020). Although the incidence of HIVAN or other glomerular diseases has declined due to the use of ART, progressive loss of kidney function is contributed by increasing comorbidities (hypertension and diabetes), co-infection with hepatitis C, drug nephrotoxicity, and also HIV-related accelerated aging (Gameiro *et al.*, 2018; Hou and Nast, 2018). A biopsy study by Kudose *et al*. revealed that glomerular disease is the predominant disease (72%) in kidney biopsy findings in PLHIV, followed by tubulointerstitial-dominant (26%) and vascular-dominant (2%). Minimal change disease (MCD) is considered a rare glomerular disease that is found in only 3 samples (1%) (Kudose *et al.*, 2020). Herein, we present a case of minimal change disease associated with HIV infection at Adam Malik General Hospital, Medan, Indonesia.

### Case Report

**Figure 1:**
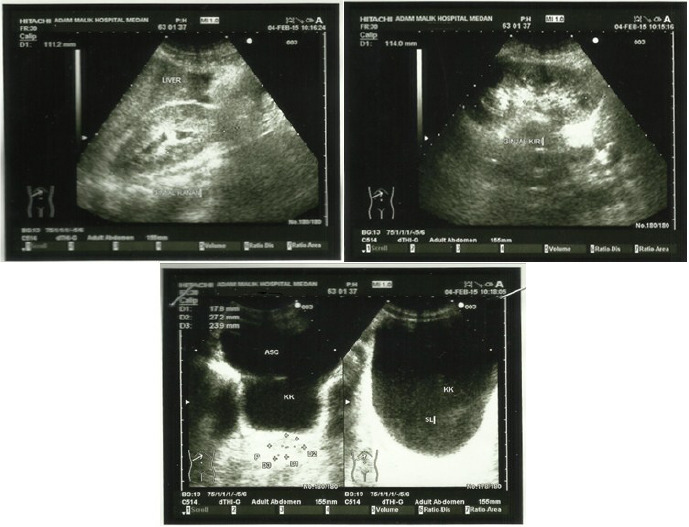
Renal and bladder ultrasonography

A 23-year-old man, Nias tribe, was admitted to Adam Malik General Hospital, Medan, with main complaints of swelling in his face, hands, feet, genitals and an enlarged abdomen for the last 2 months. The patient was homosexual, and he did not usually apply safe sex. A history of drug injection usage and blood transfusions was denied. There was no history of hypertension, and there was also no disturbance in urination. The patient complained of a markedly decreased appetite and a history of chronic diarrhea for 1 month. The nutritional status of the patient was underweight. The patient had been taking anti-tuberculosis drugs for tuberculous lymphadenitis and miliary tuberculosis. Enlarged cervical lymph nodes and generalized edema were found on physical examination. Stage 4 HIV was confirmed according to the WHO case definition. The laboratory findings were as follows: hemoglobin 11.5 g/dl, leukocytes 7,730 cell/mm^3^, platelets 205,000 cell/mm^3^, three different methods of HIV rapid test showed reactive results, absolute CD4 5 cells/µL, urea 34 mg/dl, creatinine 0.62 mg/dl, sodium 128 mEq/L, potassium 3.7 mEq/L, chloride 96 mEq/L, albumin 2.2 g/dl. Total cholesterol 83 mg/dl, triglycerides 57 mg/dl, HDL cholesterol 32 mg/dl, LDL cholesterol 40 mg/dl, HBsAg was positive, and anti-HCV was negative. Serum bilirubin, AST, ALT and alkaline phosphatase levels were normal. Rheumatoid factor and anti-dsDNA tests were negative. Urinalysis revealed +3 proteins with normal urine sediment, and no oval fat bodies were found. In the 24-hour analysis, 2.1 g of protein was found. Renal ultrasound revealed bilateral glomerulopathies with hyperechoic and cortical thickening, a normal kidney size, and ascites (Figure 1).

**Figure 2:**
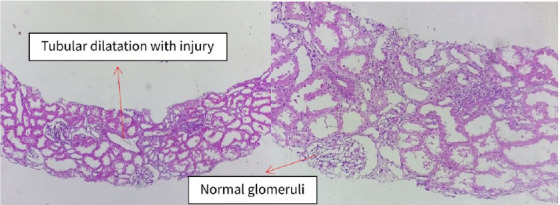
Kidney biopsy

The patient underwent a kidney biopsy, and the biopsy samples were read and interpreted at Adam Malik Hospital Medan and Gold Coast University Hospital, Australia. With hematoxylin-eosin staining under a light microscope, the glomerulus was normal, and the tubules were dilated with reduced to absence of tubular cell nuclei, implying the appearance of acute tubular injury (Figure 2). There were no cystic appearances typical of HIVAN.

The patient was treated with salt restriction, diuretics, Captopril 6.25 mg TID, Methylprednisolone 1 mg/kg BW, and combined ART, namely Tenofovir, Lamivudine, and Efavirenz, in the form of a fixed-dose combination for 2 weeks; and showed clinical improvement in edema and a decline in proteinuria. After the remission, the dose of glucocorticoids was tapered.

Two weeks after the remission, the patient came to the outpatient department with a history of a 3-day cough with rust-colored sputum, fever, malaise, and shortness of breath. The patient was lethargic, and his vital signs showed tachypnea (respiratory rate of 24 breaths/min), normal blood pressure (123/78 mmHg), an increased heart rate of 118 beats/min, a high temperature of 38.7 °C, and a decreased oxygen saturation of 88% while breathing with room air. The cardiovascular examination was unremarkable, and lung auscultation revealed bilateral rhonchi. The chest x-ray showed patchy infiltrates without cavities, suggesting pneumonia. Laboratory results showed marked leukocytosis (18,230 cells/mm^3^), thrombocytopenia (97,600 cells/mm^3^), and a raised C-reactive protein level of 117.62 mg/dL. The patient was diagnosed with sepsis associated with healthcare-associated pneumonia but was not willing to be hospitalized and passed away at home.

## Discussion

The etiology of idiopathic nephrotic syndrome in adults is mostly membranous nephropathy (30-40%), while MCD is the third most common cause (10-15%) (Bansal, 2014). As implied by the name, MCD is marked by either the absence of any glomerular abnormalities according to light microscopy or the minimal absence of immune deposits (low levels of C3 and IgM) according to immunofluorescence (Bansal, 2014; Maas *et al.*, 2016). The clinical manifestations of MCD in adults include nephrotic range proteinuria (>3.5 g/day), hypertension, hematuria, and acute renal failure (Korbet *et al.*, 1988). In contrast with MCD, HIVAN is usually found in black people with advanced HIV disease with progressive azotemia, severe proteinuria, and little or no peripheral edema (Lescure *et al.*, 2012; Silva Junior *et al.*, 2020).

The non-massive proteinuria (2.1 g/day) in this patient, accompanied by being a non-black race, made HIVAN not the first differential diagnosis before a kidney biopsy was performed. A normal glomerular appearance on the biopsy sample with concomitant tubular dilatation and the appearance of acute tubular injury (characterized by reduced to absent tubular cell nuclei) led to the diagnosis of minimal change disease. The prevalence of minimal change disease associated with HIV infection is very rare. In a study of seven renal pathologies with a total of 949 biopsies, minimal change disease was found in only 10 patients (1.05% biopsies) (Rosenberg *et al.*, 2015). Another study by Kudose *et al*. found that 3 out of 437 renal biopsies showed minimal change disease (Kudose *et al.*, 2020). This study has limitations as this is a single case report, it cannot be generalized that HIV is directly associated with minimal change disease (Nissen and Wynn, 2014), and the renal biopsy sample was analyzed by light microscopy rather than electron microscopy and immunofluorescent staining (Fogo *et al.*, 2016); therefore, the possibility of IgA nephropathy and/or IgM nephropathy could not be ruled out, and focal segmental glomerulosclerosis (FSGS) could not be eliminated if sampling errors occurred.

Cases of minimal change disease in PLHIV have been reported previously. One patient in Spain was a man with hypertension, renal insufficiency, and nephrotic syndrome whose kidney biopsy showed minimal change disease with mesangial IgA deposits (Boix *et al.*, 2000). In a cohort study of 8 patients with minimal change disease, all patients had heavy proteinuria (mean proteinuria of 7.96 g/day) (Arrestier *et al.*, 2018). Meanwhile, the patient in our case did not have all of the symptoms of nephrotic syndrome, including proteinuria that was not in the nephrotic range (only 2.1 g/day) and no hypercholesterolemia. Nephrotic syndrome without hypercholesterolemia, pseudo-nephrotic syndrome, is usually observed in lupus nephritis and diabetic nephropathy (Sukandar, 2006), but there are no reports describing pseudo-nephrotic syndrome in PLHIV.

Corticosteroid usage in PLHIV remains controversial. Corticosteroid may benefit in reducing kidney disease progression. Complete remission may occur in more than 80% of patients (Hogan and Radhakrishnan, 2013), and favorable outcomes may occur after 1.5 years of follow-up (Boix *et al.*, 2000). The use of corticosteroid in PLHIV with a CD4 cell count ≤200 cells/mm^3^ does not alter the CD4 recovery (van Welzen *et al.*, 2020). A randomized, double-blinded, placebo-controlled study by McComsey *et al*. concluded that the administration of corticosteroid within 8 weeks of the study was well tolerated and safe in PLHIV without effects on HIV RNA levels and CD4 cell count (McComsey *et al.*, 2001). Nevertheless, it is also associated with an increased risk of avascular necrosis and infection (Lucas *et al.*, 2014). In our case, the initiation of steroid therapy was discussed and decided together with the nephrologist according to the guidelines for the management of minimal change disease in the general population (Kidney Disease: Improving Global Outcomes (KDIGO) Glomerular Diseases Work Group, 2021) and by considering the risk vs benefit of corticosteroid. Antiretroviral therapy should be continued, and other supportive management, including salt restriction, ACEis or ARBs, and loop diuretics, can be utilized in managing MCD in PLHIV (Bansal, 2014). Unfortunately, therapy could not be continued in this patient because the patient had sepsis associated with healthcare-associated pneumonia, but refused to be hospitalized and eventually died.

## Conclusion

We report one case of minimal change disease in a PLHIV. Minimal change disease can occur as a renal complication of HIV infection and is usually treated effectively with steroid therapy in combination with ACE inhibitors and antiretroviral therapy. Early recognition and intervention of complications of glomerulopathy are highly important, considering that these complications can be prevented and treated. Further studies are required to provide recommendations in the field of HIV-associated renal diseases.
